# Low CD4+ T Cell Counts among African HIV-1 Infected Subjects with *Group B KIR* Haplotypes in the Absence of Specific Inhibitory KIR Ligands

**DOI:** 10.1371/journal.pone.0017043

**Published:** 2011-02-14

**Authors:** Wim Jennes, Sonja Verheyden, Christian Demanet, Joris Menten, Bea Vuylsteke, John N. Nkengasong, Luc Kestens

**Affiliations:** 1 Department of Microbiology, Institute of Tropical Medicine, Antwerp, Belgium; 2 HLA and Molecular Hematology Laboratory, Universitair Ziekenhuis Brussel (UZ Brussel), Brussels, Belgium; 3 Department of Public Health, Institute of Tropical Medicine, Antwerp, Belgium; 4 Projet RETRO-CI, Abidjan, Côte d'Ivoire; 5 Division of HIV/AIDS Prevention, National Center for HIV, STD and TB Prevention, Centers for Disease Control and Prevention, Atlanta, Georgia, United States of America; University of Toronto, Canada

## Abstract

Natural killer (NK) cells are regulated by interactions between polymorphic killer immunoglobulin-like receptors (KIR) and human leukocyte antigens (HLA). Genotypic combinations of *KIR3DS1/L1* and *HLA Bw4-80I* were previously shown to influence HIV-1 disease progression, however other *KIR* genes have not been well studied. In this study, we analyzed the influence of all activating and inhibitory KIR, in association with the known HLA inhibitory KIR ligands, on markers of disease progression in a West African population of therapy-naïve HIV-1 infected subjects. We observed a significant association between carriage of a *group B KIR* haplotype and lower CD4+ T cell counts, with an additional effect for *KIR3DS1* within the frame of this haplotype. In contrast, we found that individuals carrying genes for the inhibitory KIR ligands *HLA-Bw4* as well as *HLA-C1* showed significantly higher CD4+ T cell counts. These associations were independent from the viral load and from individual HIV-1 protective HLA alleles. Our data suggest that *group B KIR* haplotypes and lack of specific inhibitory KIR ligand genes, genotypes considered to favor NK cell activation, are predictive of HIV-1 disease progression.

## Introduction

Natural killer (NK) cells play an important role in the innate immune response against viruses and tumors, and in the regulation of the subsequent adaptive immune responses [1]. Their activity is controlled by an integration of signals from many inhibitory and activating receptors, including the killer immunoglobulin-like receptors (KIR) [2], [3]. KIR contain two or three external immunoglobulin-like domains (2D, 3D) with either long (L) or short (S) cytoplasmic tails corresponding to their function as inhibitory or activating receptors, respectively. Several inhibitory KIR have well-defined human leukocyte antigen (HLA) class I ligands. Mutually exclusive groups of HLA-C molecules with asparagine or lysine at position 80, termed C1 and C2, ligate inhibitory KIR2DL1, KIR2DL2, and KIR2DL3 [4]. A group of HLA-B molecules expressing the serologically defined Bw4 epitope recognize inhibitory KIR3DL1, with those with an isoleucine at position 80 (Bw4-80I) showing stronger inhibition than those with a threonine at this position (Bw4-80T) [5]. Both *KIR* and *HLA* loci show extreme population diversity and rapid evolution, suggesting that they are under pathogen-mediated selection and that they influence disease outcome at the individual level [6]. Indeed, several recent epidemiological studies have associated *KIR/HLA* compound genotypes with diseases as diverse as infection, autoimmune and inflammatory conditions, cancer, and reproductive failure [7].

HIV-1 infected patients show a large variation in disease courses [8]. A number of recent studies provide evidence that *KIR* and *HLA* loci play an important role in this. Flores-Villanueva et al. first found that HIV-1 patients with homozygous *Bw4* showed delayed progression to AIDS [9]. Martin et al. confirmed this but indicated that the association was derived completely from an epistatic interaction of *Bw4-80I* with *KIR3DS1*, suggesting a model in which NK cells activated through KIR3DS1 confer protection from HIV-1 disease [10]. However, subsequent studies could not confirm the *KIR3DS1/Bw4-80I* association [11], [12], and no study to date has been able to prove Bw4-80I as a true ligand for KIR3DS1 [13]–[16]. The interpretation of the *KIR3DS1/Bw4-80I* interaction was further complicated by a recent study of the same patient cohorts showing high expression alleles of inhibitory *KIR3DL1* in combination with *Bw4-80I* to also slow down disease progression [17]. Thus, it remains unclear how exactly *KIR/HLA* interactions influence HIV-1 disease outcome, and how NK cells are involved in this [18].

Few studies have analyzed *KIR* genes other than *KIR3DS1* and *KIR3DL1* in the context of HIV-1 disease. Up to 14 different functional *KIR* genes have been identified which, as a result of strong interlocus linkage disequilibrium, segregate in two broad haplotypes termed *group A* and *group B* [19], [20]. Consequently, association analyses with single *KIR* genes likely depend on the *KIR* haplotype in which they occur. In this study, we analyzed the influence of all activating and inhibitory KIR, *KIR* haplotypes, and known HLA class I inhibitory KIR ligands, on markers of disease progression in a population of West African HIV-1 infected subjects.

## Materials and Methods

### Study subjects

Eighty one HIV-1 infected female sex workers attending a confidential clinic in Abidjan, Côte d'Ivoire between January 1997 and May 2000 were studied cross-sectionally. A subset of 20 HIV-1 infected female sex workers enrolled for follow-up and paid between 2 and 4 visits to the clinic spanning a period of up to 18 months. All subjects were therapy-naïve at the time of enrolment and during follow-up.

### Ethics Statement

The study was approved by ethical committee of the Ministry of Health, Côte d'Ivoire, the ethical committee of the Institute of Tropical Medicine, Antwerp, Belgium, and by the Institutional Review Board of the Centers for Disease Control and Prevention, Atlanta, GA. All subjects gave written informed consent prior to enrolment.

### Laboratory methods

Whole blood was drawn in EDTA tubes (Becton Dickinson). Plasma was tested for HIV infection by ELISA and Western blot. CD4+ T cell counts were determined in whole blood using a FACScan flow cytometer (Becton Dickinson). HIV-1 viral load was quantified in plasma by the Amplicor HIV-1 Monitor assay, version 1.5 (Roche). Peripheral blood mononuclear cells were separated from whole blood by gradient centrifugation and stored in liquid nitrogen.

### *KIR* and *HLA class I* genotyping

Genomic DNA was extracted from peripheral blood mononuclear cells using a QIAamp DNA blood mini kit (Qiagen). *KIR* typing was performed by PCR with sequence specific primers like previously reported [21]. *KIR* haplotypes were assigned by using the current working definition available at the website of the European Bioinformatics Institute (http://www.ebi.ac.uk/ipd/kir). *Group B* haplotypes are characterized by one or more of the following genes: *KIR2DL2*, *KIR2DL5*, *KIR2DS1*, *KIR2DS2*, *KIR2DS3*, *KIR2DS5* and *KIR3DS1*. *Group A* haplotypes are characterized by the absence of all of these genes, and contain one or more of the following genes: *KIR2DL1*, *KIR2DL3*, *KIR3DL1*, and *KIR2DS4*. *KIR* genotypes consist of two *KIR* haplotypes. *AA* genotypes are identified by the absence of all *group B* haplotype genes. *AB* and *BB* genotypes are characterized by the presence of at least one *group B* haplotype gene. *AB* and *BB* genotypes cannot easily be distinguished and are collectively annotated as *Bx*. *HLA-B* and *HLA-C* typing was performed by PCR with sequence specific oligonucleotides (Gen-Probe) on a Luminex platform, which gives a DNA-based typing result at the level of 2 digits and permits to distinguish *Bw4-80I* and *Bw4-80T* from *Bw6*, and *C1* from *C2*. High resolution typing of HLA-B*58 positive samples was performed by sequencing-based typing using the AlleleSEQR *HLA-B* sequencing kit (Atria Genetics). DNA sequences were detected on an automated fluorescent DNA sequencer. *HLA-B* alleles were assigned up to six digits with the help of ASSIGN 3.5 software.

### Statistical methods

The subjects' first CD4+ T cell count and HIV-1 plasma viral load at the clinic were used in the cross-sectional analyses. The effect of single *KIR* and *HLA* genes and *KIR/HLA* combinations was assessed by linear regression analysis using log transformed CD4+ T cell counts and viral load levels as the dependent variables. Model selection was guided by the Akaike information criterion (AIC), which assesses the fit between the data and the model with a penalty for the number of parameters (i.e. favoring the more parsimonious model). Longitudinal changes in the CD4+ T cell count were examined by mixed-effects linear regression analysis with log transformed CD4+ T cell counts as the dependent variable. Statistical analyses were performed with R version 2.11.1 [22].

## Results

### Study population

HIV-1 infected female sex workers included in the study had a median age of 26 years (interquartile range (IQR), 21–32) and they reported a median duration of commercial sex work of 24 months (IQR, 12–48). At the time of enrollment, the women showed a median CD4+ T cell count of 523 cells/µl (IQR, 363–775) and a median HIV-1 plasma viral load of 4.8 log_10_ RNA copies/ml (IQR, 4.0–5.4). All subjects were therapy-naïve at the time of enrolment and during follow-up.

### *Group B KIR* haplotype genes are associated with lower CD4+ T cell counts

**Figure 1:**
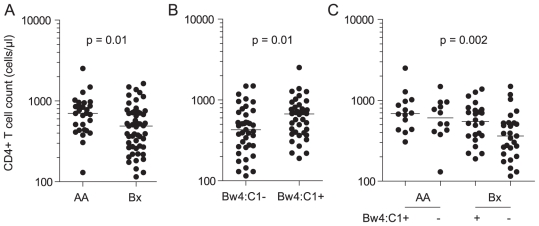
Effect of *KIR* and *HLA* genotypes on the CD4+ T cell count of HIV-1 infected subjects. (A) Effect of *AA* versus *Bx* genotype (n = 81). (B) Combined effect of inhibitory KIR ligand genes *Bw4* and *C1* (n = 73). *Bw4:C1* denotes the simultaneous occurrence of Bw4 and C1. (C) Combined effect of *Bx* and *Bw4:C1* genotypes (n = 73). P values represent the statistical significance of the linear regression models using log-transformed CD4+ T cell counts as the dependent variable. Horizontal lines represent median values.

First we investigated whether the extensive variability in *KIR* gene content was associated with the CD4+ T cell count or plasma viral load of the HIV-1 infected subjects (Table 1a). The presence of *KIR2DL2*, *KIR2DL5*, *KIR2DS3*, and *KIR3DS1* was associated with significantly lower CD4+ T cell counts. These genes are all specific to the *group B KIR* haplotype and, accordingly, subjects with a *KIR Bx* genotype (i.e., those carrying one or two *group B* haplotypes) showed a significantly lower CD4+ T cell count. No such associations were found for the viral load, although trends were seen for a number of *group B KIR* haplotype genes mirroring the effects on the CD4+ T cell count. To investigate whether individual *group B* haplotype genes showed effects on the CD4+ T cell count beyond that of the *Bx* genotype, we analyzed them together by multivariate linear regression (Table 2a). Only the addition of *KIR3DS1* resulted in a better fit of the model relative to that with *Bx* alone (AIC of 156 vs. 158). These data show that HIV-1 infected subjects carrying a *group B KIR* haplotype have lower CD4+ T cell counts (Fig. 1A), and even more so if they also have *KIR3DS1* within the frame of this haplotype.

**Table 1:** Univariate effects of *KIR* and *HLA* genes on the CD4+ T cell count and HIV-1 viral load level of 81 HIV-1 infected subjects.

	Frequency n (%)	CD4+ T cell count			HIV-1 viral load		
		Fold difference (CI)	AIC	p	Fold difference (CI)	AIC	p
**a. KIR genes and genotypes**							
2DL1	77 (95)	1.46 (0.75–2.82)	163	0.260	4.27 (0.60–30.4)	202	0.144
2DL2	48 (59)	0.70 (0.53–0.93)	158	**0.015**	1.36 (0.56–3.30)	204	0.485
2DL3	63 (78)	1.07 (0.76–1.51)	164	0.702	1.13 (0.40–3.20)	204	0.811
2DL5	45 (56)	0.74 (0.56–0.98)	160	**0.039**	1.99 (0.84–4.71)	202	0.114
3DL1	80 (99)	0.54 (0.15–1.98)	163	0.345	10.0 (0.21–480)	203	0.239
2DS1	9 (11)	1.00 (0.63–1.59)	164	0.990	0.91 (0.23–3.59)	204	0.892
2DS2	42 (52)	0.78 (0.58–1.03)	161	0.077	0.98 (0.41–2.33)	204	0.961
2DS3	25 (31)	0.72 (0.53–0.97)	160	**0.031**	1.59 (0.63–4.03)	203	0.323
2DS4	79 (98)	1.14 (0.45–2.87)	164	0.784	9.75 (0.64–149)	201	0.101
2DS5	24 (30)	0.81 (0.59–1.10)	162	0.171	1.64 (0.64–4.19)	203	0.300
3DS1	5 (6.2)	0.51 (0.29–0.92)	159	**0.025**	1.96 (0.33–11.7)	204	0.454
AA	28 (35)	1.47 (1.10–1.96)	158	**0.010**	0.61 (0.25–1.53)	203	0.290
Bx	53 (65)	0.68 (0.51–0.91)	158	**0.010**	1.63 (0.65–4.04)	203	0.290
**b. KIR ligand genes**							
Bw4	63 (78)	1.24 (0.88–1.74)	163	0.222	0.90 (0.32–2.53)	204	0.835
Bw6	56 (69)	1.07 (0.78–1.46)	164	0.671	1.01 (0.40–2.58)	204	0.981
Bw4-80I	58 (72)	1.19 (0.87–1.64)	163	0.274	1.00 (0.38–2.60)	204	1.000
Bw4-80T	7 (8.6)	1.08 (0.65–1.81)	164	0.761	0.69 (0.15–3.20)	204	0.632
C1^a^	52 (71)	1.31 (0.94–1.83)	146	0.105	0.98 (0.35–2.76)	188	0.972
C2	66 (81)	0.97 (0.67–1.41)	164	0.880	0.87 (0.29–2.63)	204	0.798
**c. HLA alleles**							
B*57	3 (3.7)	1.63 (0.77–3.47)	163	0.200	0.28 (0.03–2.73)	203	0.272
B*58:01	10 (13)	0.99 (0.64–1.54)	163	0.959	1.29 (0.33–5.06)	201	0.711

Data are calculated by linear regression analysis using log transformed CD4+ T cell counts and HIV-1 viral load levels as the dependent variables. CI, 95% confidence interval; AIC, Akaike information criterion for goodness of fit; p, statistical significance.

a data available for 73 of 81 subjects. p values<0.05 are in bold type.

**Table 2:** Multivariate effects of *KIR* and *HLA* genes on the CD4+ T cell count of 81 HIV-1 infected subjects.

	Term 1		Term 2		Term 3			Model
	Fold difference (CI)	p	Fold difference (CI)	p	Fold difference (CI)	*P*	AIC	p
**a. ** ***KIR*** ** combinations**								
Bx+2DL2	0.75 (0.41–1.38)	0.358	0.89 (0.50–1.61)	0.703			159	0.035
Bx+2DL5	0.68 (0.41–1.12)	0.127	1.00 (0.62–1.62)	0.983			160	0.037
Bx+2DS2	0.65 (0.42–1.02)	0.061	1.05 (0.69–1.61)	0.813			159	0.036
Bx+2DS3	0.74 (0.53–1.04)	0.078	0.83 (0.59–1.17)	0.286			158	0.021
Bx+2DS5	0.69 (0.50–0.96)	0.030	0.97 (0.68–1.36)	0.840			159	0.037
Bx+3DS1	0.72 (0.54–0.96)	0.025	0.58 (0.34–1.03)	0.063			156	0.007
**b. ** ***HLA*** ** combinations** a								
Bw4+C1	1.48 (1.00–2.19)	0.049	1.46 (1.04–2.05)	0.030			144	0.038
Bw4+Bw4:C1	1.02 (0.66–1.56)	0.945	1.46 (1.04–2.05)	0.030			144	0.038
C1+Bw4:C1	0.98 (0.64–1.52)	0.945	1.48 (1.00–2.19)	0.049			144	0.038
Bw4:C1	1.47 (1.10–1.97)	0.010					142	0.010
**c. ** ***KIR/HLA*** ** interactions** a								
2DL2+C1+2DL2:C1	0.76 (0.43–1.36)	0.349	1.35 (0.78–2.32)	0.277	0.92 (0.46–1.82)	0.797	145	0.056
2DL3+C1+2DL3:C1	1.12 (0.55–2.29)	0.756	1.26 (0.60–2.66)	0.535	1.06 (0.46–2.44)	0.891	150	0.343
2DS2+C1+2DS2:C1	0.59 (0.34–1.04)	0.071	1.04 (0.62–1.73)	0.880	1.41 (0.73–2.74)	0.305	146	0.076
2DS3+C1+2DS3:C1	0.53 (0.31–0.92)	0.024	0.96 (0.62–1.50)	0.854	1.76 (0.89–3.46)	0.102	145	0.049
**d. ** ***KIR/HLA*** ** combinations** a								
Bx+Bw4:C1+Bx:Bw4:C1	0.63 (0.41–0.98)	0.041	1.29 (0.79–2.10)	0.297	1.18 (0.65–2.15)	0.585	140	0.005
Bx+Bw4:C1	0.69 (0.51–0.93)	0.015	1.44 (1.09–1.91)	0.012			138	0.002
Bx+3DS1+Bw4:C1	0.73 (0.54–0.97)	0.033	0.53 (0.29–0.98)	0.044	1.39 (1.05–1.84)	0.021	136	<0.001

Data are calculated by multivariate linear regression analysis using log transformed CD4+ T cell counts as the dependent variable. The estimated effects of the respective terms in the models are shown. Interaction terms, denoting the simultaneous occurrence of two genes, are annotated with “:”.

a Data available for 73 of 81 subjects. CI, 95% confidence interval; AIC, Akaike information criterion for goodness of fit; p, statistical significance.

### Inhibitory KIR ligand genes *HLA-Bw4* and *HLA-C1* are associated with higher CD4+ T cell counts

Next, we investigated whether inhibitory KIR ligand genes were associated with the CD4+ T cell count or plasma viral load of the HIV-1 infected subjects. Subjects with *Bw4* or *C1* showed a trend towards higher CD4+ T cell counts, with no effects on the viral load (Table 1b). Interestingly, the effects of *Bw4* and *C1* on the CD4+ T cell count became statistically significant when combined in a multivariate model (Table 2b). Addition of a *Bw4:C1* interaction term, denoting the simultaneous occurrence of *Bw4* and *C1*, abrogated the individual effects of *Bw4* and *C1*, indicating that they were derived from subjects harboring both genes. On its own, the *Bw4:C1* interaction term explained the variation in CD4+ T cell counts best. These data show that HIV-1 infected subjects carrying inhibitory KIR ligand genes *Bw4* as well as *C1* have higher CD4+ T cell counts than those lacking either *Bw4* or *C1* (Fig. 1B). Two member alleles of *Bw4*, B*57 and B*58:01, are known to display individual HIV-1 protective effects in African populations [23]. In our population, B*57 showed slightly higher CD4+ T cell counts and slightly lower viral load levels while no such effects were seen for B*58:01 (Table 1c). Adjustment for both B*57 and B*58:01 did little to abrogate the observed effects of *Bw4* (1.21-fold; 95% CI, 0.85–1.74; p = 0.285) or *Bw4:C1* (1.47-fold; 95% CI, 1.06–2.03; p = 0.045), suggesting that *Bw4* acts at least in part independently from B*57 and B*58:01.

### *KIR* and *HLA* genes have both synergistic and independent effects on the CD4+ T cell count

The protective effects of *C1* and *Bw4* on the CD4+ T cell counts could result from its known interactions with *KIR*. To test this, we investigated whether their effects could be abrogated by addition of *KIR:HLA* interaction terms to the multivariate linear regression models (Table 2c). Functional studies have shown that C1 binds to inhibitory KIR2DL2 and KIR2DL3, while Bw4 binds to inhibitory KIR3DL1 [4], [5], [24]. C1 and Bw4 have also been suggested to ligate activating KIR2DS2/KIR2DS3 and KIR3DS1, respectively, but this could not be confirmed to date [13]–[16], [25]. We could not detect statistical interaction of *C1* with *KIR2DL2* or *KIR2DL3*: the interaction terms did not abrogate the *C1* effects. However, trends towards statistical interaction were observed for *C1* with *KIR2DS2* and *KIR2DS3*: in both cases the interaction terms showed protective effects at the cost of *C1*. These models indicate that the negative effects of *KIR2DS2* and *KIR2DS3* are dampened in the presence of *C1* (*KIR2DS2*: 0.59-fold in the absence versus 0.83-fold in the presence of *C1*; *KIR2DS3*: 0.53-fold in the absence versus 0.93-fold in the presence of *C1*), and that this capacity explains most of the protective effect of *C1*. Because of the low numbers of subjects with *KIR3DS1* or without *KIR3DL1* in our study population, we were not able to calculate interactions of *Bw4* with *KIR3DL1* or *KIR3DS1*.

Next, we investigated whether the best-fitting *KIR* and *HLA* combinations were independent of each other by analyzing them together in multivariate linear regression models (Table 2d). Overall, the effects of *Bx* and *Bw4:C1* genotypes were found to be independent, with no signs of interaction. Consequently, a better fit of the data can be expected for combinations of these genotypes. Indeed, the combination of *Bx* and *Bw4:C1* showed a major increase in fit relative to that of *Bw4:C1* alone, and even more so if *KIR3DS1* was added to the model (AIC values of 142, 138 and 136, respectively). The additive effects of *Bx* and *Bw4:C1* are shown in Fig. 1C, with HIV-1 patients possessing a *KIR Bx* genotype in the absence of *Bw4:C1* showing the lowest CD4+ T cell counts.

### Carriage of a *Bx* genotype in absence of *Bw4:C1* is associated with faster CD4+ T cell decline

**Figure 2:**
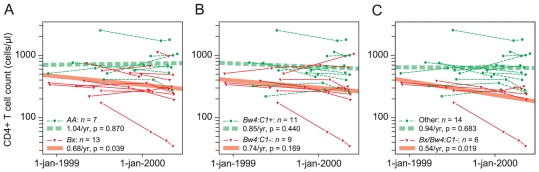
Effect of *KIR* and *HLA* genotypes on the rate of CD4+ T cell decline among HIV-1 infected subjects. Thin lines represent individual CD4+ T cell count profiles (n = 20). Thick lines represent the fitted models calculated by mixed-effects linear regression analysis. (A) Effects of *AA* and *Bx* genotypes. (B) Effect of *Bw4:C1* genotype. (C) Effect of *Bx* genotype in the absence of *Bw4:C1*. *Bw4:C1* denotes the simultaneous occurrence of *Bw4* and *C1*. The estimated fold decrease in CD4+ T cell count per year and p values are shown.

The low CD4+ T cell counts associated with a *Bx* genotype in absence of *Bw4:C1* could result from a higher rate of CD4+ T cell decline during acute and/or chronic infection, or simply from a longer total duration of infection, a distinction that cannot be made by cross-sectional analyses. The total duration of infection is not available for this population of HIV-1 seroprevalent female sex workers, but it can be estimated by the total duration of commercial sex work which constitutes the main risk factor for HIV-1 infection in this population. No differences in duration of commercial sex work were observed between subjects with or without *Bx* (median values of 25 vs 22 months, p = 0.392) or *Bw4:C1* (median value of 24 months for both groups, p = 0.588) and neither did adjustment for the duration of commercial sex work affect the associations between *Bx* or *Bw4:C1* and the CD4+ T cell count (*Bx*: 0.68-fold; 95% CI, 0.51–0.91; p = 0.010; *Bw4:C1*: 1.46-fold; 95% CI, 1.08–1.97; p = 0.013). Next, we analyzed longitudinal changes in the CD4+ T cell count for a subset of 20 HIV-1 infected female sex workers with available follow up by mixed-effects linear regression analysis (Fig. 2). Subjects with a *Bx* genotype showed a statistically significant decline in CD4+ T cell count; this was not found for subjects with an *AA* genotype. Lack of *Bw4:C1* did not result in a statistically significant decline in CD4+ T cell count on its own, but when combined with the presence of a *Bx* genotype, it provided the strongest effect. Together, these analyses suggest that the observed associations between *Bx* and *Bw4:*C1 and the CD4+ T cell count do not result from differences in the time since infection but from differences in the rate of CD4+ T cell decline during follow-up.

## Discussion

Genotypic combinations of *KIR3DS1/L1* and *HLA Bw4-80I* were previously shown to influence HIV-1 disease progression, however other *KIR* genes have not been well studied. Therefore, we analyzed all activating and inhibitory KIR, in association with the known inhibitory KIR ligands, in a population of West African HIV-1 infected subjects.

We first found that HIV-1 infected subjects with a *group B KIR* haplotype showed markedly lower CD4 counts and faster CD4 count decline than those without. Significant differences were observed for the individual *group B* haplotype genes *KIR2DL2*, *KIR2DL5*, *KIR2DS3* and *KIR3DS1*, with *KIR3DS1* showing an additional effect to that of the *group B* haplotype. These data confirm previous findings from Gaudieri et al., who found HIV-1 disease promoting effects for several *group B* haplotype genes including *KIR3DS1* [11], and from Martin et al. who noted faster disease progression among carriers of *KIR3DS1* in the absence of *HLA Bw4-80I* [10].

Next, we found that HIV-1 infected subjects carrying genes for both Bw4 and C1 showed significantly higher CD4+ T cell counts. This is in agreement with several previous reports showing associations between *Bw4* or *Bw4-80I* and protection from HIV-1 disease progression [9], [10], [26], [27]. No previous study observed a protective effect for *C1*. Recent studies identified a *−35C* single nucleotide polymorphism near *HLA-C* that was associated with HIV-1 control and increased expression of HLA-C [28], [29], however this variant was not associated with either group of *C1* or *C2* alleles, nor could its effects be explained by one or more individual *HLA-C* alleles [29], [30]. Similarly, in our study, *C1* did not preferentially contain *HLA-C* alleles in known linkage with *−35C* (data not shown). Remarkably, in our study, the protective effects of *Bw4* and *C1* appeared to be completely derived from subjects harboring both genes. This could reflect the known linkage disequilibrium between several *HLA-B* and *HLA-C* alleles and their observed joint effects on HIV-1 control [31]–[33].

Interestingly, we noted a trend towards statistical interaction of *C1* with *KIR2DS2* and *KIR2DS3*, which explained most of the *C1* effect and resulted in a weakening of the negative effects of *KIR2DS2* and *KIR2DS3* in the presence of *C1*. No statistical interaction could be detected between *C1* and inhibitory *KIR2DL2* or *KIR2DL3*, despite in vitro evidence of C1 binding to KIR2DL2/3 but not KIR2DS2/3 [4], [24], [25]. It is plausible, however, that the interaction of *C1* with *KIR2DL2/3* appeared neutral because only 2 subjects in our study population lacked both *KIR2DL2* and *KIR2DL3*, and thus that the presence of *C1* was the only limiting factor in establishing the effect. Hence, we speculate that the weakening of the negative effects of *KIR2DS2* and *KIR2DS3* by *C1* is mediated by its interaction with *KIR2DL2/3*. This conclusion concurs with previous reports showing a link between *KIR2DS1* and *KIR2DS2* and risk for developing psoriatic arthritis, particularly in the absence of the HLA ligands for their homologous inhibitory receptors, KIR2DL1 and KIR2DL2/3 [34], [35]. Previously, Martin et al observed a similar statistical interaction between *Bw4-80I* and *KIR3DS1* influencing HIV-1 disease progression, however, in contrast to the tempering effects noted in the present study, the *Bw4-80I/KIR3DS1* interaction term completely inversed the negative effects of *KIR3DS1* now resulting in significant protection from disease progression [10]. Unfortunately, subsequent studies could not confirm this epistatic *Bw4-80I/KIR3DS1* interaction [11], [12], and in the present study we did not have sufficient numbers of subjects with *KIR3DS1* to replicate these calculations.

*Group B KIR* haplotypes are considered to be more activating than *group A* haplotypes due to the higher numbers of activating *KIR* genes that they contain [3], [6]. Indeed, subjects with a *KIR AB* genotype were shown to display higher NK cell responsiveness than those with an *AA* genotype [36]. On the other hand, HLA Bw4 and C1/2 ligands for inhibitory KIR provide the necessary inhibitory signals that set the NK cell activation threshold and guarantee self-tolerance [3], [6]. Consequently, our data suggest that *KIR* and *HLA* genotypes that favor NK cell activation, like carriage of a *group B KIR* haplotype and/or lack of a Bw4 or C1 inhibitory KIR ligand, play a detrimental role in the progression of HIV-1 disease. This conclusion is at variance with previous reports by Martin et al, in which a protective role for activated NK cells in HIV-1 disease progression was proposed [10], [17]. In these studies, *KIR3DS1/Bw4-80I* genotypes were suggested to directly activate NK cells [10], while strongly inhibitory *KIR3DL1/Bw4-80I* genotypes were hypothesized to display greater NK cell activation capacity due to better NK cell licensing [17]. Indeed, subsequent in vitro studies were able to show stronger NK-cell mediated virus inhibition, functional capacity, and specific NK cell subset expansion during acute HIV-1 infection for these genotypes [37]–[39]. However, other studies found that Bw4 strongly inhibited proliferation and cytotoxicity of KIR3DL1-expressing NK cells without any influence on KIR3DS1 [16], and that possession of KIR3DS1 alone was sufficient to induce strong NK cell effector functions [40]. In fact, we believe that our interpretation of a detrimental role for activating *KIR/HLA* genotypes makes more sense in the light of emerging data on innate immune activation during acute HIV-1 infection in establishing HIV-1 pathogenesis. For instance, acute SIV infection of non-progressing sooty mangabeys is characterized by substantially reduced levels of innate immune activation, including lower IFN-α production, IFN-regulated gene expression, and NK cell proliferation, compared to that seen in AIDS progressing rhesus macaques [41], [42]. Like rhesus macaques, HIV-1 infected humans show significant innate immune activation and expansion of NK cells during the acute phase of the infection [43], [44]. In agreement with this, a recent study found only low levels of antiviral NK cell activity together with a low frequency of *KIR3DS1* in a group of rare HIV-1 controller patients [45]. Although the possible pathogenic mechanisms of carrying an activating *KIR/HLA* genotype remain unclear, studies have shown direct NK cell-mediated killing of CD4+ T cells that express NKp44L, a ligand for NKp44 that is induced upon exposure to a HIV-1 gp41, to correlate with HIV-1 disease progression [46], [47]. Alternatively, CD4+ and CD8+ T cells are known to express KIR and could be involved as well.

Interestingly, we found that *KIR/HLA* genotypes correlated best with the CD4+ T cell count and not, or at least much more weakly, with the viral load of the study subjects. This is in agreement with a previous *KIR* study noting better correlations with disease outcomes involving the CD4+ T cell count than the viral load [11]. In part, this could reflect the lower within-patient variation for the CD4+ T cell count, and the fact that the CD4+ T cell count shows a better clinical prognostic value than the viral load, especially during the chronic phase of the infection (the disease stage most of our study subjects are expected to be in) [48]. However, preferential correlations with the CD4+ T cell count could also reflect the specific immune responses that are involved, with activating *KIR/HLA* genotypes that drive cytotoxic responses being expected to directly impact on target cell numbers. In that respect, our data are in agreement with studies of SIV-infected primate species showing a strong link between rapid control of innate immune activation and lack of CD4 count depletion or disease progression, however without any influence on the often high levels of viral replication seen in these animals [41], [42]. In this model, we could hypothesize a role for viral load at the beginning of the causal pathway, with viral peptides modulating the interactions of activating and inhibitory KIR with their ligands, within the context of the inherited *KIR/HLA* genotype [49], [50].

Previously, we found that carriage of an *AB KIR* genotype and of certain inhibitory *KIR* combinations in the absence of their Bw4 or C1 ligands was associated with resistance to HIV-1 infection in the same population of African female sex workers [51]. These findings corroborated previous data of increased NK cell-mediated cytotoxicity in HIV-1 resistant drug users [52], and have since then been confirmed by several other studies showing phenotypic and/or genotypic activating KIR profiles in HIV-exposed seronegative populations [53]–[55]. Interestingly, the genotypes observed in our previous study are nearly the same as the ones identified here, despite differences in study design and data analysis (e.g., all *AB* genotypes in our previous study are actually *Bx* according to current guidelines, and combinations of *KIR2DL2* and *KIR2DL3* coincide with the presence of a *AA* or *Bx* genotype). Together, this means that in our population of African female sex workers, similar activating KIR/HLA genotypes are associated with resistance to HIV infection as well as with faster progression of the disease. This is paradoxical, yet could be logically explained by an intriguing model in which an activated NK cell profile protects the host against HIV-1 acquisition through rapid and potent elimination of HIV-1 infected target cells, but once systemically infected, promotes HIV-1 disease progression through over-activation of the innate and adaptive arms of the immune system.

The observed associations between *KIR/HLA* genotype and CD4+ T cell count in this study were detected by a cross-sectional analysis of therapy-naïve HIV-1 infected female sex workers. This approach is less sensitive than a longitudinal analysis of time to AIDS and it could be biased by the unknown duration of infection of the included subjects. To address this, we first used the total duration of commercial sex work, the main risk factor for HIV-1 infection in this population, as an estimate of the time since infection, and found that adjustment had no impact on the observed associations. Secondly, we calculated the longitudinal effects of the *KIR/HLA* genotypes on the rate of CD4+ T cell decline in a subgroup of female sex workers with available follow-up. This analysis confirmed the cross-sectional associations, however it was rather limited in number of included subjects as well as in number of follow-up samples. Thus, confirmation of our findings in larger longitudinal cohorts of untreated HIV-infected subjects remains warranted. Furthermore, future functional studies should investigate whether activating *KIR* profiles are expressed on NK cells, CD8+ T cells, or both, and to what extent the activating *KIR/HLA* genotypes proposed in this study translate into increased in vivo immune activation and enhanced in vitro target cell cytotoxicity.

In summary, we found that *group B KIR* haplotypes and lack of specific inhibitory KIR ligand genes, genotypes considered to favor NK cell activation, are associated with CD4+ T cell loss during HIV-1 infection. Better understanding of how genetic variation at *KIR* and *HLA* loci influences HIV-1 pathogenesis may lead to the development of immune intervention strategies aiming at controlling progression of the disease.
